# Validation of imaging reporting and data system of coronavirus disease 2019 lexicons CO-RADS and COVID-RADS with radiologists’ preference: a multicentric study

**DOI:** 10.1186/s43055-021-00485-2

**Published:** 2021-04-15

**Authors:** Haisam Atta, Hosam A. Hasan, Reham Elmorshedy, Adel Gabr, Wael A. Abbas, Mohamed M. El-Barody

**Affiliations:** 1 Department of Radiology, South Egypt Cancer Institute, Assiut University, Assiut, 71515 Egypt; 2 Department of Radiology, Faculty of Medicine, Assiut University, Assiut, Egypt; 3 Chest Department, Faculty of Medicine, Assiut University, Assiut, Egypt; 4 Department of Medical Oncology, South Egypt Cancer Institute, Assiut University, Assiut, Egypt; 5 Department of Internal Medicine, Faculty of Medicine, Assiut University, Assiut, Egypt

**Keywords:** COVID-19, Pandemics, Pneumonia, Viral, Tomography, X-Ray computed

## Abstract

**Background:**

A retrospective multicentric study gathered 1439 CT chest studies with suspected coronavirus disease 2019 (COVID-19) affection. Three radiologists, blinded to other results, interpreted all studies using both lexicons with documentation of applicability and preferred score in assessing every case. The purpose of the study is to assess COVID-19 standardized assessment schemes’ (CO-RADS and COVID-RADS lexicons) applicability and diagnostic efficacy.

**Results:**

This study included 991 RT-PCR-confirmed CT studies. An almost perfect agreement was found in COVID-RADS among the three observers (Fleiss Kappa = 0.82), opposed by a substantial agreement in CO-RADS (*Κ* = 0.78). The preference records favor COVID-RADS/CO-RADS in 78.5%/12.5%, 75.5%/24.5%, and 73.4%/24.5% regarding the three radiologists’ records, respectively. The distinguishability between positive and negative RT-PCR cases was 0.92 for COVID-RADS, while it was 0.85 for CO-RADS. On the other hand, both lexicons’ performance regarding clinical diagnosis and clinical suspicion index was 0.93 for COVID-RADS and 0.94 for CO-RADS. A very high to excellent agreement between the three observers for COVID-RADS/CO-RADS preference was concluded (Fleiss Kappa = 0.80 to 0.94). These results were statistically significant (*p* < 0.001).

**Conclusion:**

Both lexicon scores (CO-RADS and COVID-RADS) were found to be applicable in the COVID-19 structured report with the preference of COVID-RADS in more than 50% of cases. The diagnostic accuracy of COVID-RADS against RT-PCR was higher than that of CO-RADS.

## Background

Since the global coronavirus disease (COVID-19) emerged in late December 2019 and the declaration of a pandemic by the World Health Organization (WHO) in March 2020, the mystery regarding its cause, origin, mode of spread, and vaccination efficacy rose, with the infection of more than 88 million confirmed patients and 1.9 million deaths until the first week of January 2021 [1–3]. There are suggestions that the COVID-19 situation will not resolve shortly, on the progress of a second wave worldwide, and its virulence with different postulations of occurrence of variants and mutations [4, 5].

The standard reference for COVID-19 diagnosis is reverse transcriptase-polymerase chain reaction (RT-PCR) due to its high sensitivity, but it has a lengthy turnaround time that may reach up to 72 h. In addition to the fact that multiple negative RT-PCR test results may be needed to exclude the presence of disease in the setting of a high clinical suspicion [6].

Imaging has a leading role in solving the disease’s ambiguity through its application in diagnosis, management, and follow-up of cases. Some countries have even established imaging as an essential first-line diagnostic test [7].

To strengthen the radiology’s role in the pandemic emergency, the radiological report should be tailored to provide clear communication with the referring clinicians [8]. Preferably to be free of ambiguity to facilitate comparison of results and guide appropriate patient care. This can be achieved easily by employing a structured report form linked to a well-defined scoring system simulating a reporting and data system (RADS) lexicon [9].

Few different standardized assessment schemes were developed for COVID-19 pulmonary involvement, among them the CO-RADS lexicon [10], developed by the Dutch radiological society, and the COVID-RADS lexicon, which was postulated by Salehi et al. [11]. The authors recommended that empirical data studies should validate their proposals. To the best of our knowledge, a single study by Inui et al. [12] handled the diagnostic performance and compared the different grading systems of COVID-19 chest CT findings.

The current study aims to assess both lexicons’ (CO-RADS and COVID-RADS) applicability in structural reporting of COVID-19 affection by assessing both scores’ diagnostic performance and estimating the interobserver reliability, performance, and preference agreement among both lexicon scores.

## Methods

### Study design and patient population

This is a retrospective multicentric study; thus, ethical approval was issued from the local ethical committee, waving the need to receive informed consent from the patients due to the study’s nature.

From February 2020 to July 2020, 1439 consecutive CT chest studies with suspected COVID-19 affection were gathered from four different imaging centers, three in Upper Egypt and one in West of Saudi Arabia (KSA).

**Fig. 1:**
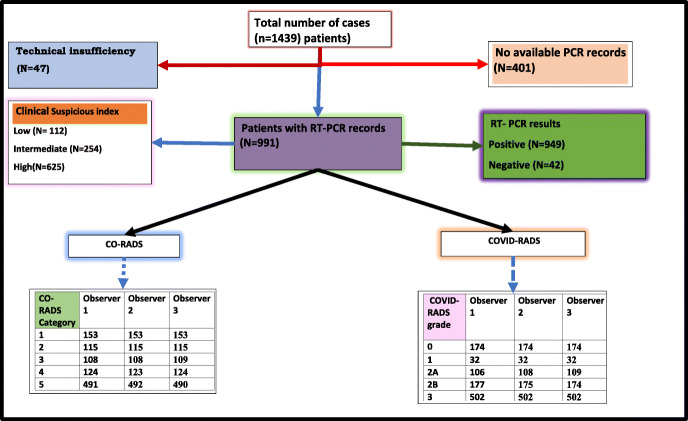
CONSORT participant flow diagram

A total of 448 studies were excluded: 47 studies were excluded due to technical insufficiency to perform a proper interpretation or assign a score and 401 studies due to deficient RT-PCR results (Fig. 1).

To confirm the sample size efficiency, sample size calculation was carried out using G*Power 3 software [13]. A calculated minimum sample of 900 studies was needed to detect an effect size of 0.1 in the interrater reliability of the COVID-RADS and CO-RADS, with an error probability of 0.05 and 95% power on a two-tailed test.

### Image acquisition and interpretation

CT scans covered an area from the root of the neck down to the infra-diaphragmatic region. No intravenous contrast was used.

CT imaging protocols from different vendors were employed, and imaging parameters acquired were summarized in Table 1.

**Table 1:** Technical parameters employed in chest imaging within the multiple centers

	First center	Second center	Third center	Fourth center
Vendor	*Philips*	*Philips*	Toshiba	GE
Model	Ingenuity	Brilliance	Lightning acquilion	Bright speed
Number of detectors	32	64	16	16
Scout				
kV	100	120	120	120
mAs	30	30	50	10
Scan				
kV	120	120	100–120	120
mAs	300	100	Auto mA 100	Auto mA 100
Pitch	0.798	0.6	1.1	1.375
Gantry tilt	0	0	0	0
FOV	Depends on body size			

Studies were revised on a dedicated workstation using window width (WW) 1500 and window level (WL) − 400 for the lung window and 450 WW and 60 WL for the mediastinal window.

### Lexicon score implementation

Three radiologists revised the CT images: two senior radiologists with 20 years of experience and a younger radiologist with 14 years of experience. Radiologists were blinded to the other interpretation results and did not have access to the RT-PCR results at the time of interpretation.

CT findings were categorized as atypical, fairly typical, or typical findings suggesting COVID-19 affection. A combination of typical or atypical findings was also categorized according to the proposed coronavirus disease 2019 (COVID-19) imaging reporting and data system and RSNA Consensus Statement regarding Chest CT findings of COVID-19 [7, 11, 14–19].

**Fig. 2:**
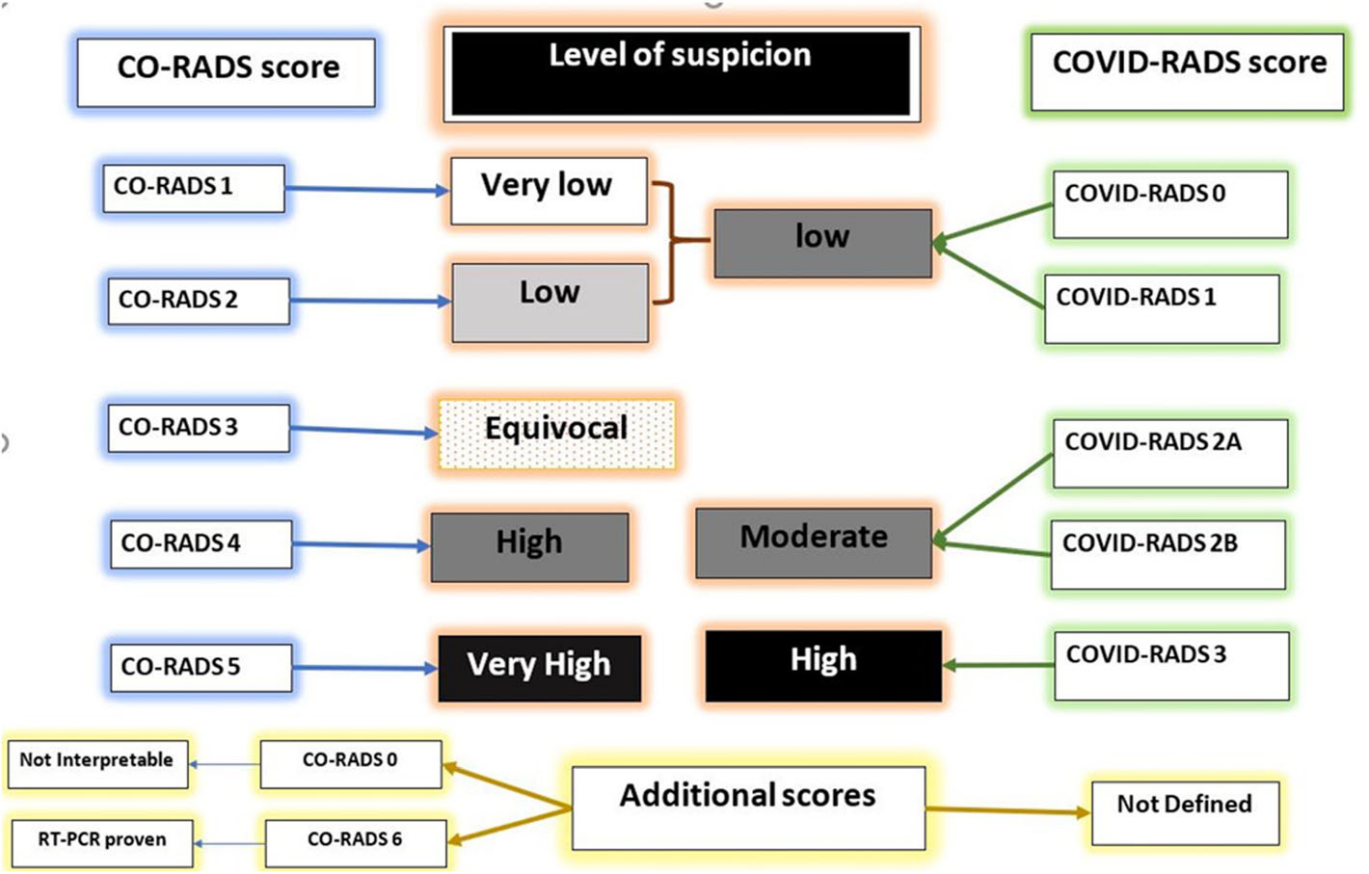
Different levels of COVID-19 suspicion and correspondent score in both lexicons

**Fig. 3:**
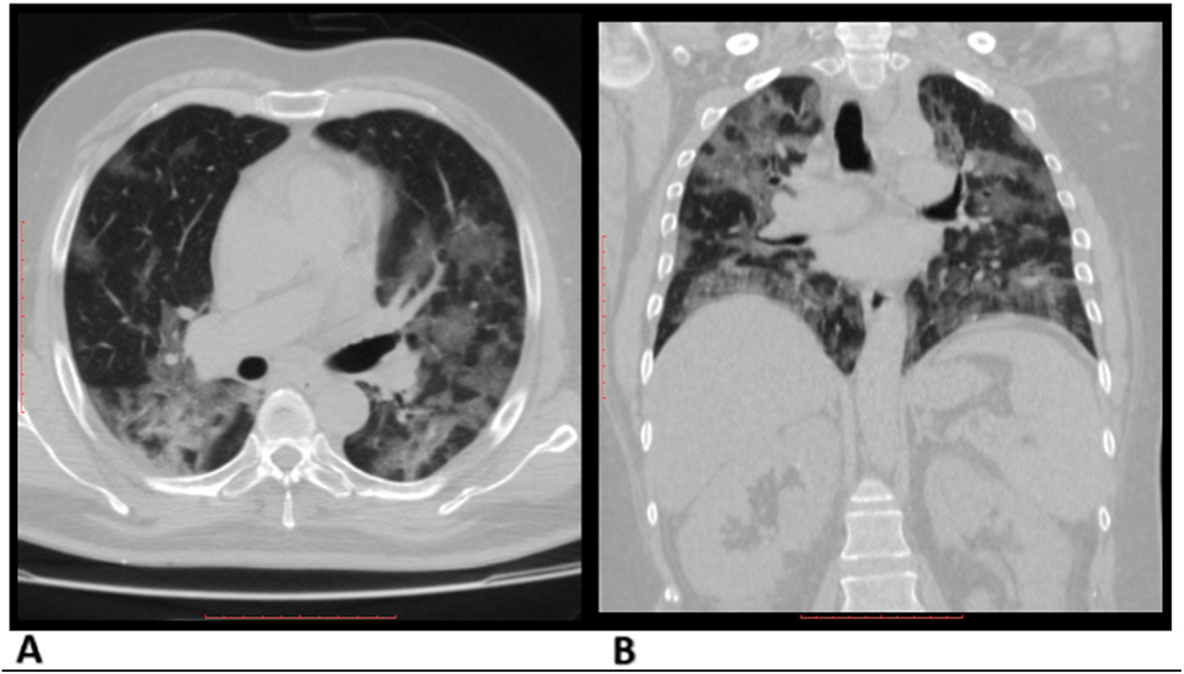
CT typical findings of COVID-19 affection. A 48-year-old man with multiple ground-glass lesions and a crazy-paving pattern. Observers agreed on COVID-RADS score 3 and CO-RADS score 5. The preference of COVID-RADS: CO-RADS was (2:1). RT-PCR result was positive

**Fig. 4:**
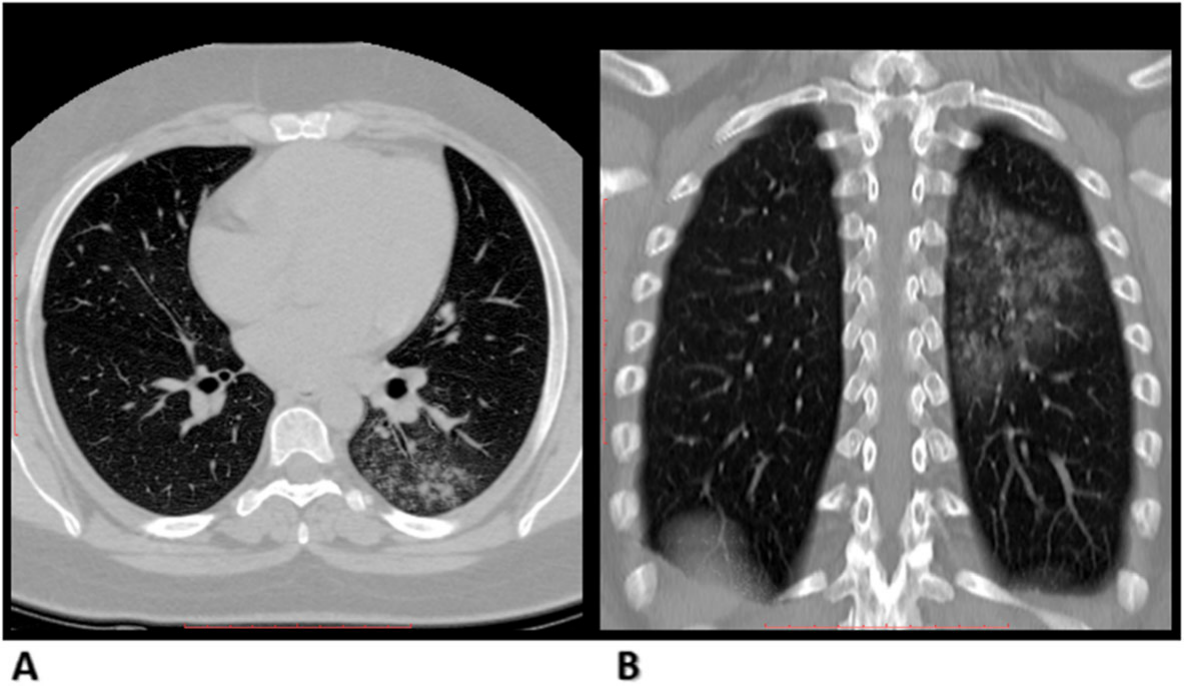
A 23-year-old woman with CT findings of consolidation in the left lower lung lobe with the tree on bud appearance Observers agreed on COVID-RADS score 1 and CO-RADS score 2. The preference of COVID-RADS: CO-RADS was (1:2). RT-PCR result was negative

**Fig. 5:**
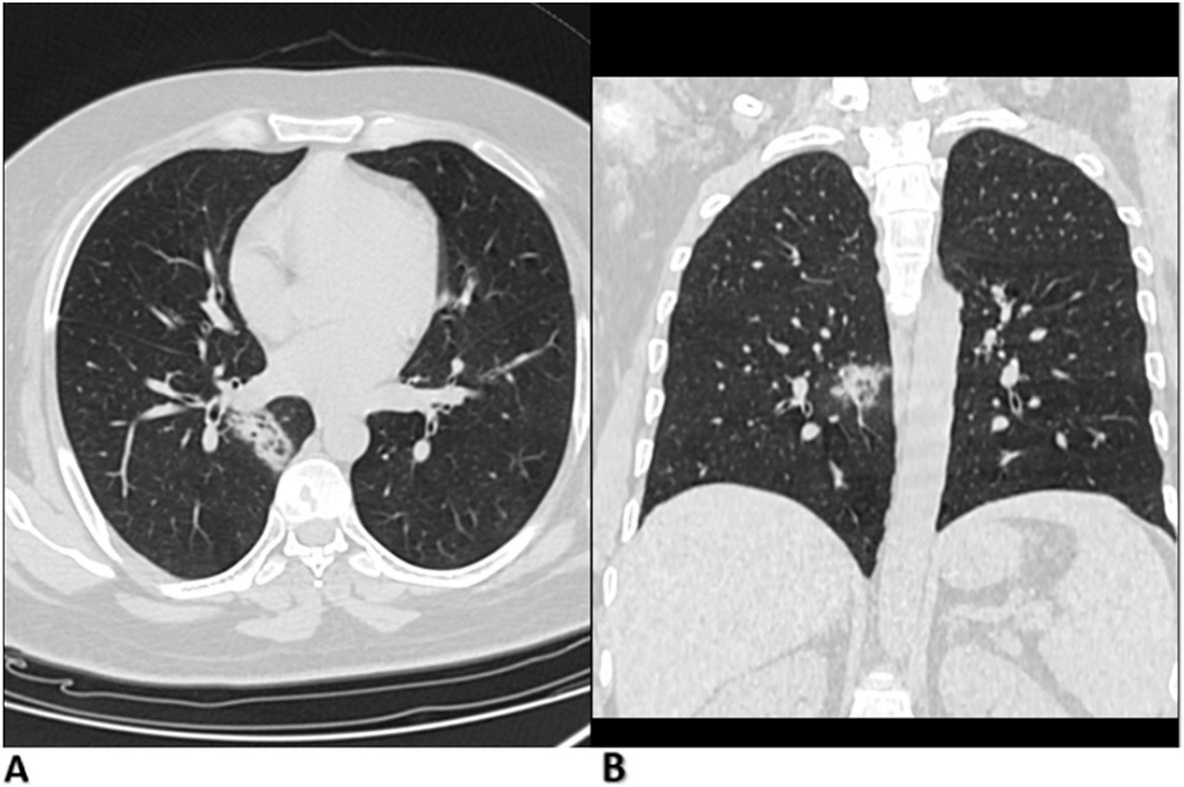
A 36-year-old man with CT findings of single ground-glass opacity. Observers interpreted 3 and 4 CO-RADS scores and agreed on COVID-RADS score 2A. The preference was in favor of COVID-RADS to CO-RADS (3:0). RT-PCR result was positive

After categorizing these findings, radiologists applied both lexicon scores, CO-RADS [10] and COVID-RADS scores [11], for each case and translated the lexicon scores to different equivalent levels of suspicion of COVID-19 affection (Fig. 2). Radiologists performed a case-based score, evaluated the ease of applicability on applying each score, and documented which score was preferred in assessing this particular case (Figs. 3, 4, and 5).

We omitted CO-RADS zero score in our assessment, as we already excluded cases with technical insufficiency (*n* = 47). Moreover, we did not include CO-RADS 6 score as the RT-PCR assay results were not revealed during interpretation. This omission facilitated the comparative study of both lexicons as these CO-RADS scores, 0 and 6, are not included in the COVID-RADS lexicon that did not include correspondent scores (Fig. 2)

### Statistical analysis

Data were verified, coded, and analyzed by the researcher, using SPSS version 24. Fleiss’ Kappa extension from the Extension Hub in SPSS Statistics was used, and then Fleiss’ Kappa analysis was carried out using the Fleiss’ Kappa procedure. Descriptive statistics: means, standard deviations, medians, interquartile range (IQR), and percentages were calculated. Fleiss’ Kappa was calculated to assess the reliability of agreement between readers. Kappa characteristics of CO-RADS and COVID-RADS of each observer were compared to the median of the other observer. The area under the curve (AUC) of the receiver operating characteristic curve (ROC) for each observer was given and separated from the reference standards defined by RT-PCR alone and RT-PCR together with clinical diagnosis. Spearman’s ranked correlation was employed to assess the correlation between symptom to imaging interval and the clinical and imaging assessment results. *P* value < 0.05 was considered significant.

## Results

### Patients and characteristics of the studied cohort

Nine hundred ninety-one cases are the number of eligible cases after excluding 448 studies with total readings of 2973 studies. Positive RT-PCR results were encountered in 949 patients (95.7% of total cases), and negative RT-PCR was recorded in 42 (4.2%) patients (Fig. 1).

Eight hundred ninety-two patients (90%) were Egyptian, while 99 (9.9%) were Saudi among our included patients; their age indices were 44.82 ± 16.1 years (mean ± SD). The cohort included 558 male (56.3%) and 433 female (43.7%) patients.

**Fig. 6:**
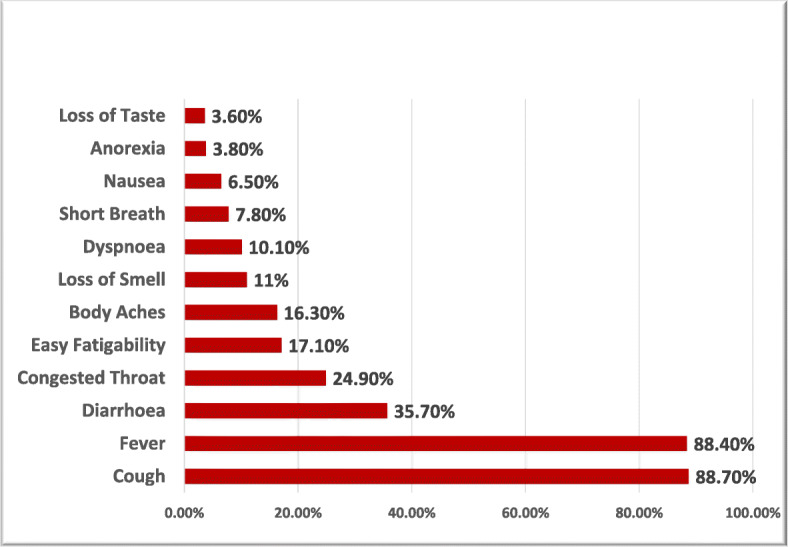
Prevalence of clinical symptoms among the study patient’s cohort

The main presenting symptoms were cough and fever in 879 (88.7%) cases. Diarrhea was among the most common presenting symptoms as it was encountered in 354 (35.7%) patients, more than 1/3 of our sample size, while anosmia was among the least presenting symptom, observed in 109 (11%) patients of our patient cohort (Fig. 6). The clinical suspicion index of COVID infection was high in 625 (63.1%) patients and intermediate in 254 (25.6%) patients, while 112 (11.3%) patients showed a low clinical suspicion index; the RT-PCR has been repeated in 177 (17.9%) cases and was conclusive from the first time in 814 (82%) patients [20].

**Fig. 7:**
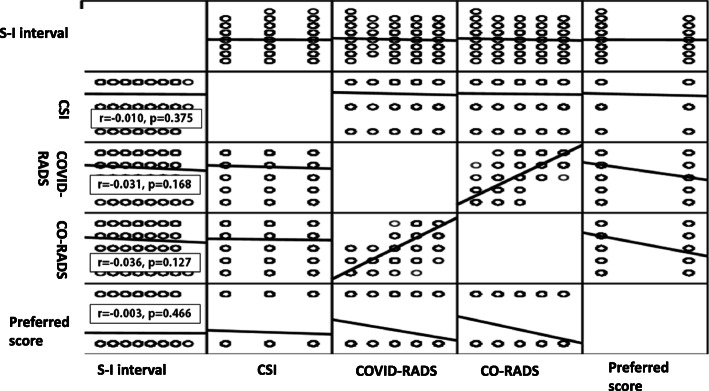
Correlation matrix for the symptoms to imaging duration interval (S-I interval) versus clinical suspicion index (CSI) and assessment of both lexicon score results (CO-RADS or COVID-RADS) or even the preferred score among COVID-19 patients

Figure 7 showed that there was no/minimal insignificant negative correlation between symptoms to imaging interval (S-I Interval) vs. clinical suspicion index (CSI) and assessment of both lexicon score results (CO-RADS or COVID-RADS) or even the preferred score among COVID-19 patients (*r* = − 0.003 to − 0.036, *p* > 0.05).

### Lexicon score performance concerning the relation with different levels of COVID-19 affection

In comparing both lexicon performance, radiologists applied both of them for every single case with feasibility of 100% for each lexicon.

There was an absolute agreement in 2124 readings (71% of cases) for COVID-RADS, while with CO-RADS, the agreement was confined to only 27% of cases (804 readings) (Fig. 1 and Table 2). This was seen with a perfect agreement in COVID-RADS among the three observers (*K* = 0.82). On the other hand, there is a substantial agreement for CO-RADS among the three observers’ overall reliability (*Κ* = 0.78).

**Table 2:** Diagnostic findings according to observers and different levels of suspicion of COVID affection

Parameter (*n* = 991)	Category	Observer 1	Observer 2	Observer3
o COVID_RADS results	Low	206 (20.8%)	206 (20.8%)	206 (20.8%)
	Moderate	283 (28.6%)	283 (28.6%)	283 (28.6%)
	High	502 (50.6%)	502 (50.6%)	502 (50.6%)
o COVID_RADS feasibility	Feasible	968 (97.7%)	968 (97.7%)	968 (97.7%)
o CO-RADS results	Very low	153 (15.4%)	153 (15.4%)	153 (15.4%)
	Low	115 (11.6%)	115 (11.6%)	115 (11.6%)
	Equivocal	108 (10.9%)	108 (10.9%)	109 (10.9%)
	High	124 (12.5%)	123 (12.4%)	124 (12.5%)
	Very high	491 (49.5%)	492 (49.6%)	490 (49.4%)
CO-RADS feasibility	Feasible	910 (91.8%)	915 (92.3%)	915 (92.3%)
o Observer preference	COVID-RADS	778 (78.5%)	748 (75.5%)	727 (73.4%)
	CO-RADS	213 (21.5%)	243 (24.5%)	264 (26.6%)

COVID-RADS grades showed an absolute agreement among the three observations within grades zero, one, and three. A slight disagreement was encountered in grades 2A and 2B (in an average of 2–3 cases).

On the other hand, the difference in interpretation among the three observers when handling CO-RADS lexicon was in one case in CO-RADS scores 3 and 4, while disagreement in the documentation of CO-RADS score 5 was observed in two cases.

These agreement issues were similar in translating both lexicon scores into a suspicion index (Fig. 2 and Table 2).

### Interobserver reliability, performance, and preference agreement

The different observers recorded that the COVID-RADS lexicon was applicable in 97.7% of cases, while CO-RADS categorization was applicable in 91.8% for the first observer, while it was applicable in 92.3% of cases in the records of second and third observers.

The observer performance favored the COVID-RADS lexicon with 78.5%, 75.5%, and 73.4% for the three radiologists involved in the study. In comparison, CO-RADS performance was 21.5%, 24.5%, and 26.6%, respectively.

Distinguishing the ability between positive and negative RT-PCR cases was 0.92 (92% CI: 0.85–0.96) for COVID-RADS, while this distinguishability was 0.85 (85% CI: 0.87–0.99) for CO-RADS. On the other hand, both lexicons’ performance regarding clinical diagnosis and clinical suspicion index was 0.93 (93% CI: 0.87–0.99) for COVID-RADS and 0.94 (94% CI: 0.90–0.99) for CO-RADS.

Table 3 illustrates the interobserver reliability and performance regarding COVID-RADS/CO-RADS. For COVID-RADS, it was found that there was a very high reliability of observer 1 compared with the other two (observer 1 vs. observers 2 and 3; Fleiss Kappa = 0.89). Likewise, there was a high reliability of observer 2 compared with the other two observers (Fleiss Kappa = 0.78). Also, there was a high reliability of observer 3 compared with the other two observers (Fleiss Kappa = 0.75). The overall reliability of the three observers was very high (Fleiss Kappa = 0.82). Regarding CO-RADS, there was very high reliability of observer 1 compared with the others (observer 1 vs. observers 2 and 3; Fleiss Kappa = 0.86). Likewise, there was a very high reliability of observers 2 and 3 in comparison (Fleiss Kappa = 0.91). The overall reliability of the three observers was high (Fleiss Kappa = 0.78).

**Table 3:** Interobserver reliability and performance

Observer no.	*Κ* value^a^	AUC^b^ vs. RT-PCR	AUC vs. clinical suspicion index
COVID-RADS			
Observer 1	0.89 (0.84–0.98)	0.94 (0.87–0.98)	0.95 (0.93–0.99)
Observer 2	0.78 (0.84–0.92)	0.91 (0.78–0.97)	0.91 (0.85–0.98)
Observer 3	0.75 (0.84–0.87)	0.89 (0.74–0.95)	0.90 (0.84–0.97)
Overall	0.82 (0.84–0.91)	0.92 (0.85–0.96)	0.93 (0.87–0.99)
CO-RADS			
Observer 1	0.86 (0.64–0.97)	0.88 (0.81–0.97)	0.93 (0.88–0.99)
Observer 2	0.91 (0.77–0.99)	0.84 (0.79–0.93)	0.92 (0.87–0.98)
Observer 3	0.91 (0.77–0.99)	0.81 (0.75–0.92)	0.89 (0.82–0.96)
Overall	0.78 (0.59–0.91)	0.85 (0.76–0.94)	0.94 (0.90–0.99)

^a^Fleiss *Κ* value and 95% CI (confidence interval)

^b^AUC (area under the curve)

The interobserver preference agreement (Table 4) showed the agreement levels between observers regarding COVID-RADS/CO-RADS preference.

**Table 4:** Interobserver preference agreement

		Observer 1		*Κ* value^a^
		COVID-RADS	CO-RADS	
Observer 2	COVID-RADS	738 (74.5%)	10 (1%)	0.86 (0.69–0.95)
	CO-RADS	40 (4%)	203 (20.5%)	*P* < 0.001
Observer 3	COVID-RADS	716 (72.3%)	11 (1.1%)	0.80 (0.61–0.93)
	CO-RADS	62 (6.3%)	202 (20.4%)	*P* < 0.001
		Observer 2		*Κ* value^a^
		COVID-RADS	CO-RADS	
Observer 3	COVID-RADS	726 (73.3%)	1 (0.1%)	0.94 (0.79–0.99)
	CO-RADS	22 (2.2%)	242 (24.4%)	*P* < 0.001

^a^Fleiss *Κ* value and 95% CI (confidence interval)

Our results declared very high agreement between the three observers for COVID-RADS/CO-RADS preference (observer 1 vs. observers 2 and 3; Fleiss Kappa = 0.86 and 0.80, respectively). Likewise, there was an excellent agreement between observers 2 and 3 (Fleiss Kappa = 0.94). These results were statistically significant (*p* < 0.001).

## Discussion

Chest CT has a high sensitivity for diagnosing COVID-19 [21]. Scientific societies have different recommendations for the employment of imaging in COVID-19 management, with many recommendations against CT’s employment in screening. To enhance the role of imaging and CT chest, structural reporting is established to improve communication between radiologists and clinicians with recommendations to include a RADS system among the declared systems since the start of the global pandemic, among the popular lexicons are CO-RADS and COVID-RADS [21].

Both lexicons were created to categorize the suspicion with COVID-19 affection, whereas the typical CT findings were graded as CO-RADS score 5 and COVID-RADS score 3 with the implication of very high and high suspicion level according to each lexicon, respectively. On the other hand, normal chest CT findings are categorized as CO-RADS score 1 or COVID-RADS score 0, reflecting unlikely occurrence or low suspicion, but both scores are not an exclusion of COVID-19 affection [10, 11].

Moreover, the atypical findings are consistent with COVID-RADS score 1 and CO-RADS score 2, implicating a low level of suspicion in both lexicons. Furthermore, the COVID-RADS lexicon included score 2A that includes fairly atypical findings or score 2B that combines typical/fairly typical and atypical findings, while CO-RADS score 4 includes suspicious abnormalities with a high level of suspicion [10, 11].

CO-RADS score 3 reflects an indeterminate level of suspicion. Such a score is not included in the COVID-RADS lexicon [10, 11].

The CO-RADS lexicon contains grade 0 for technically insufficient studies and grade 6 for positive RT-PCR cases [10]. The latter two grades were not included in our study to facilitate comparison between both lexicons as COVID-RADS does not include opposing categories.

The clinical picture of COVID-19 disease is quite variable [22]. Among our patient cohorts, cough, fever, and diarrhea were the most common presenting symptoms, to the point that some authors considered feco-oral transmission as a potential transmission route [23]. While Menni et al. [24] have reported that loss of taste and smell senses as pathognomonic symptoms, the current study recorded them among the least presenting symptom, this in agreement with Gautier et al. [25].

The majority of cases had a high clinical suspicion index as the study was carried out during the pandemic phase. This may also explain the rush to perform CT imaging in the disease’s early phases [18].

Sultan et al. [26] documented a significant difference in pulmonary CT findings of COVID-19 with the variation in clinical presentation onset duration. Ding et al. [27] defined 6 stages of different durations in the course of the disease. Accordingly, Prokop et al. [10] subjects fell in the second and third groups. In comparison, the current study cohort was categorized in the first two stages.

The early tendency to perform imaging could be explained by the fact that in developing countries, CT imaging may be considered the only available diagnostic modality due to the shortage of laboratory kits facing a spike in patient numbers or even logistic strains; developed countries are not an exclusion from these circumstances [24, 28].

Despite that, Ding et al. [27] reported that disease findings change rapidly at the early stages. However, the current study findings indicated no/minimal correlation was present between symptoms to imaging duration interval versus clinical suspicion index or assessment of both lexicon scores results (CO-RADS or COVID-RADS) or even the preferred score. On the other hand, Pan et al. [29] concluded that the greatest affection likely occurs after 10 days from symptoms initiation.

Comparing both lexicon performance, almost perfect agreement in COVID-RADS was found among the three observers (*K* = 0.82). On the other hand, A substantial agreement with the three observers’ overall reliability (*Κ* = 0.78); similar results were also reported in Prokop et al. [10]. Also, Inui et al. [12] reported that both CO-RADS and COVID-RADS provided a reasonable agreement in COVID-19 reporting of chest CT findings.

On the other hand, Prokop et al. documented that the indeterminate category CO-RADS-3 offered little diagnostic efficacy as a declaration of the COVID pandemic. This could explain that the COVID-RADS lexicon did not include a grade for indeterminate lesions.

In the current study, radiologists’ recorded that both lexicons feasibility are 100% with possible assignment in all cases, and both lexicons are easily applied in more than 90% of cases according to their interpretation among the different levels of COVID-19 affection. Moreover, the observers preferred COVID-RADS with a higher percentage in more than 50% of the cases.

We attribute this preference to the fact that COVID-RADS includes clear CT findings for the different typical and atypical findings. Also, employing this lexicon is done by revising, checking the criteria, and ticking a checklist of each case, hence detecting the grade and reflecting the suspicion of viral infection, besides the proper organization of the COVID-RADS lexicon as its postulation was based on an evidence-based systemic review.

In contrast, the Dutch group developed the CO-RADS score in the pandemic’s acute stage with rapidly increasing cases and resource restrictions. This was acknowledged among their limitations [10].

Among this study’s advantages are the multicentric enrollment with different exposure levels to the COVID-19 pandemic to formulate a representative sample from four different centers to COVID affection.

Although the radiologists’ experiences are very close with narrow differences, we did not face significant differences in interpretation, and this confirms the applicability of both scores and endorses the employment of the RADS lexicon within a structural report.

Few limitations face the current study: the retrospective nature of the study, the non-inclusion of the severity score of lung affection, and the unavailability of the median interval between imaging and RT-PCR.

On facing the global emergency of COVID-19, we recommend employing a structured report form to fully facilitate the interpretation and improve communication with referring clinicians to conquer the time factor in the management of suspected COVID patients, basing the final diagnosis on clinical, laboratory, and imaging findings and finally a confirmed RT-PCR assay.

## Conclusion

In conclusion, both lexicon scores (CO-RADS and COVID-RADS) are applicable in the COVID-19 structured report with the preference of COVID-RADS in more than 50% of cases. The diagnostic accuracy of COVID-RADS against RT-PCR is 92%, while that of CO-RADS is 85%.

## Data Availability

The datasets used and/or analyzed during the current study are available from the corresponding author on reasonable request.

## Abbreviations

AUC: Area under the (ROC) Curve CI Confidence interval COVID-19 Coronavirus disease 2019 CT Computerized tomography KSA Kingdom of Saudi Arabia RAD Reporting and data system ROC Receiver operating characteristic RSNA Radiological Society of North America RT-PCR Reverse transcription-polymerase chain reaction WL Window level WW Window width
